# Macrophage-biomimetic porous Se@SiO_2_ nanocomposites for dual modal immunotherapy against inflammatory osteolysis

**DOI:** 10.1186/s12951-021-01128-4

**Published:** 2021-11-22

**Authors:** Cheng Ding, Chuang Yang, Tao Cheng, Xingyan Wang, Qiaojie Wang, Renke He, Shang Sang, Kechao Zhu, Dongdong Xu, Jiaxing Wang, Xijian Liu, Xianlong Zhang

**Affiliations:** 1 Department of Orthopaedics, Shanghai Jiao Tong University Affiliated Sixth People’s Hospital, Shanghai Jiao Tong University, Shanghai, 200233 China; 2 College of Chemistry and Chemical Engineering, Shanghai University of Engineering Science, Shanghai, 201620 China

**Keywords:** Biomimetic nanoparticle, Porous Se@SiO_2_ nanospheres, Macrophage polarization, Osteolysis, Immunomodulation

## Abstract

**Background:**

Inflammatory osteolysis, a major complication of total joint replacement surgery, can cause prosthesis failure and necessitate revision surgery. Macrophages are key effector immune cells in inflammatory responses, but excessive M1-polarization of dysfunctional macrophages leads to the secretion of proinflammatory cytokines and severe loss of bone tissue. Here, we report the development of macrophage-biomimetic porous SiO_2_-coated ultrasmall Se particles (porous Se@SiO_2_ nanospheres) to manage inflammatory osteolysis.

**Results:**

Macrophage membrane-coated porous Se@SiO_2_ nanospheres(M-Se@SiO_2_) attenuated lipopolysaccharide (LPS)-induced inflammatory osteolysis via a dual-immunomodulatory effect. As macrophage membrane decoys, these nanoparticles reduced endotoxin levels and neutralized proinflammatory cytokines. Moreover, the release of Se could induce macrophage polarization toward the anti-inflammatory M2-phenotype. These effects were mediated via the inhibition of p65, p38, and extracellular signal-regulated kinase (ERK) signaling. Additionally, the immune environment created by M-Se@SiO_2_ reduced the inhibition of osteogenic differentiation caused by proinflammation cytokines, as confirmed through in vitro and in vivo experiments.

**Conclusion:**

Our findings suggest that M-Se@SiO_2_ have an immunomodulatory role in LPS-induced inflammation and bone remodeling, which demonstrates that M-Se@SiO_2_ are a promising engineered nanoplatform for the treatment of osteolysis occurring after arthroplasty.

**Graphical Abstract:**

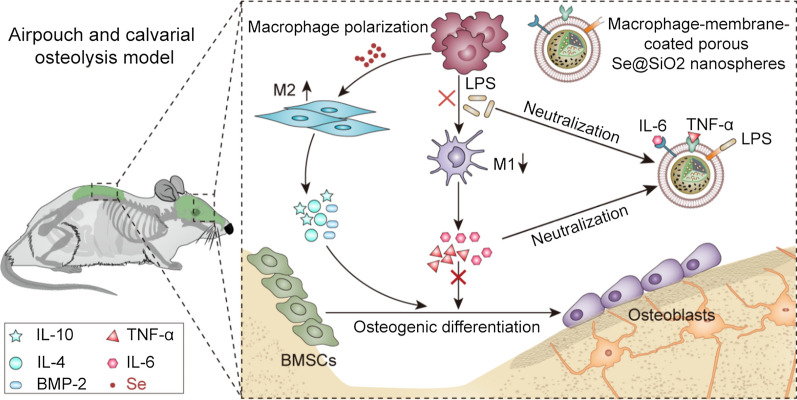

**Supplementary Information:**

The online version contains supplementary material available at 10.1186/s12951-021-01128-4.

## Background

For end-stage diseases that affect the joints, total joint replacement is the most common and successful surgical treatment method. However, following replacement therapy, aseptic loosening and periprosthetic joint infection are serious complications that can cause inflammatory osteolysis, affecting the continued use of the prosthesis, necessitating revision surgery and imposing a heavy economic burden [1, 2]. Inflammatory osteolysis is induced by bacterial products and/or implant-derived wear particles, which activate innate immune cells to produce proinflammatory factors that can disrupt osteogenic processes and destroy bone [3–5].

Macrophages are key effector immune cells in inflammatory osteolysis and are generally considered to have two polarization states following activation, namely, the M1 and M2 phenotypes. Following exposure to certain inflammatory stimuli, excessive polarization of macrophages toward the proinflammatory M1 phenotype is accompanied by the secretion of large amounts of proinflammatory cytokines, such as interleukin (IL)-6 and tumor necrosis factor-alpha (TNF-α), which enhance osteoclast activity and impair the osteogenic process [6, 7]. In contrast, M2-type macrophages are induced by IL-4 or transforming growth factor-beta; they secrete IL-10 and IL-4 to inhibit inflammation and promote tissue repair and functional recovery [8, 9]. Several studies have demonstrated that M2 macrophages have important roles in bone immunology and that an appropriate anti-inflammatory immune response attenuates bone tissue damage while facilitating the osteogenic process [10–12]. Hence, avoiding excessive M1-polarization and promoting M2-polarization are critical in reducing bone tissue loss and promoting repair. Se is an essential trace element for the human body, as it plays key roles in nutrition, physiology, pathology, and disorder treatments [13]. Studies have shown that Se has antioxidative, anti-inflammatory, and regulatory functions in immune cells [14–16]. Moreover, Se can regulate macrophage phenotypes and improve the anti-inflammatory function of macrophages to promote tissue repair and cell proliferation [17]. However, its relatively narrow range between an effective concentration and toxicity limits the application of Se-containing drugs [18]. Hence, we previously developed porous SiO_2_-coated ultrasmall Se particles (Se@SiO_2_ nanospheres) for drug delivery, which slowly release effective Se at effective concentrations, thereby reducing toxicity and improving biocompatibility [19].

Lipopolysaccharide (LPS), an endotoxin derived from the cell wall of gram-negative bacteria, is an effective inducer of immune cells and a key factor in the occurrence of inflammatory osteolysis [20, 21]. It promotes M1 polarization through membrane-bound toll-like receptor 4 (TLR4) and downstream nuclear factor-κB (NF-κB) and mitogen-activated protein kinase (MAPK) signaling pathways, resulting in the secretion of proinflammatory cytokines [22]. In recent years, engineered nanoparticles coated with cell membranes have been increasingly used in cancer treatment, disease diagnosis, antibacterial therapy, and detoxification [23–26]. Cell membrane-based nanoplatforms are characterized by phagocytosis evasion, prolonged circulation, and effective targeting [27–29]. Moreover, the membranes of various cells, such as red blood cells, macrophages, platelets, and tumor cells, can be used according to different functional requirements [30–32]. For instance, macrophage membrane-coated nanoparticles have been used to treat sepsis, rheumatoid arthritis and induce bone regeneration owing to their ability to neutralize endotoxins and proinflammatory cytokines, providing a promising delivery system for nanotherapeutics against inflammatory osteolysis [33–35]. Macrophage membrane-coated nanoparticles exhibit the same characteristic antigenic properties as macrophages, with membrane protein receptor conservation, indicating their potential to bind to inflammatory mediators and block inflammatory responses. In light of the above findings, reducing the production of inflammatory stimuli and regulating macrophage polarization are two potential approaches to inhibit osteolysis.

**Scheme 1:**
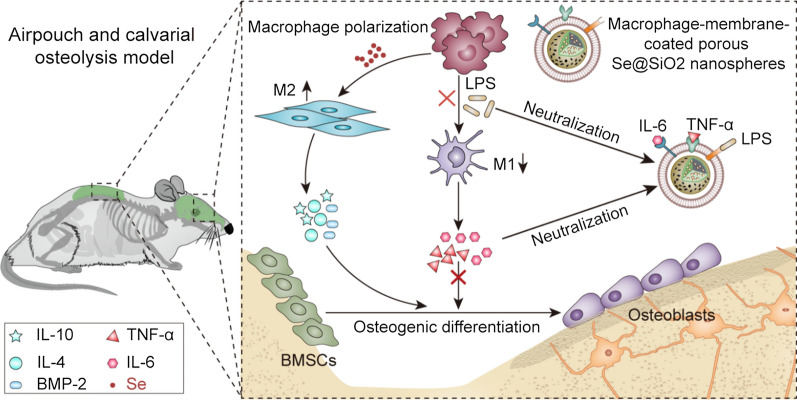
Macrophage membrane-coated porous Se@SiO_2_ nanospheres(M-Se@SiO_2_) attenuate lipopolysaccharide (LPS)-induced inflammatory osteolysis by modulating macrophage polarization toward an anti-inflammatory phenotype, reducing toxin levels, and neutralizing inflammatory cytokines

In this study, we established a drug delivery system based on macrophage membrane-coated porous Se@SiO_2_ nanospheres (M-Se@SiO_2_). Macrophage membranes express key protein receptors such as TNF-R, IL6-R, and TLR4, which bind to the inflammatory cytokines TNF-α, IL-6, and endotoxin LPS, respectively, to inhibit M1 polarization. Simultaneously, porous Se@SiO_2_ nanospheres release Se to induce the polarization of macrophages toward the anti-inflammatory M2-phenotype, thereby reducing excessive proinflammatory activity and promoting osteogenesis. These effects may be mediated via the inhibition of p65, p38, and extracellular signal-regulated kinase (ERK) signaling. Additionally, the immunomodulatory effect of macrophages on osteogenic differentiation was investigated. M-Se@SiO_2_ reduced the inhibition of osteogenic differentiation caused by inflammatory cytokines. This study provides a dual immunomodulatory strategy to treat inflammatory osteolysis. The biomimetic membrane system reduces toxin levels and neutralizes inflammatory cytokines, and Se released from nanoparticles regulates the polarization of macrophages. The findings showed that M-Se@SiO_2_ may serve as a new tool for influencing immunomodulatory osteogenesis in the disease microenvironment (Scheme 1).

## Results and discussion

### Characterization of M-Se@SiO_2_

**Fig. 1:**
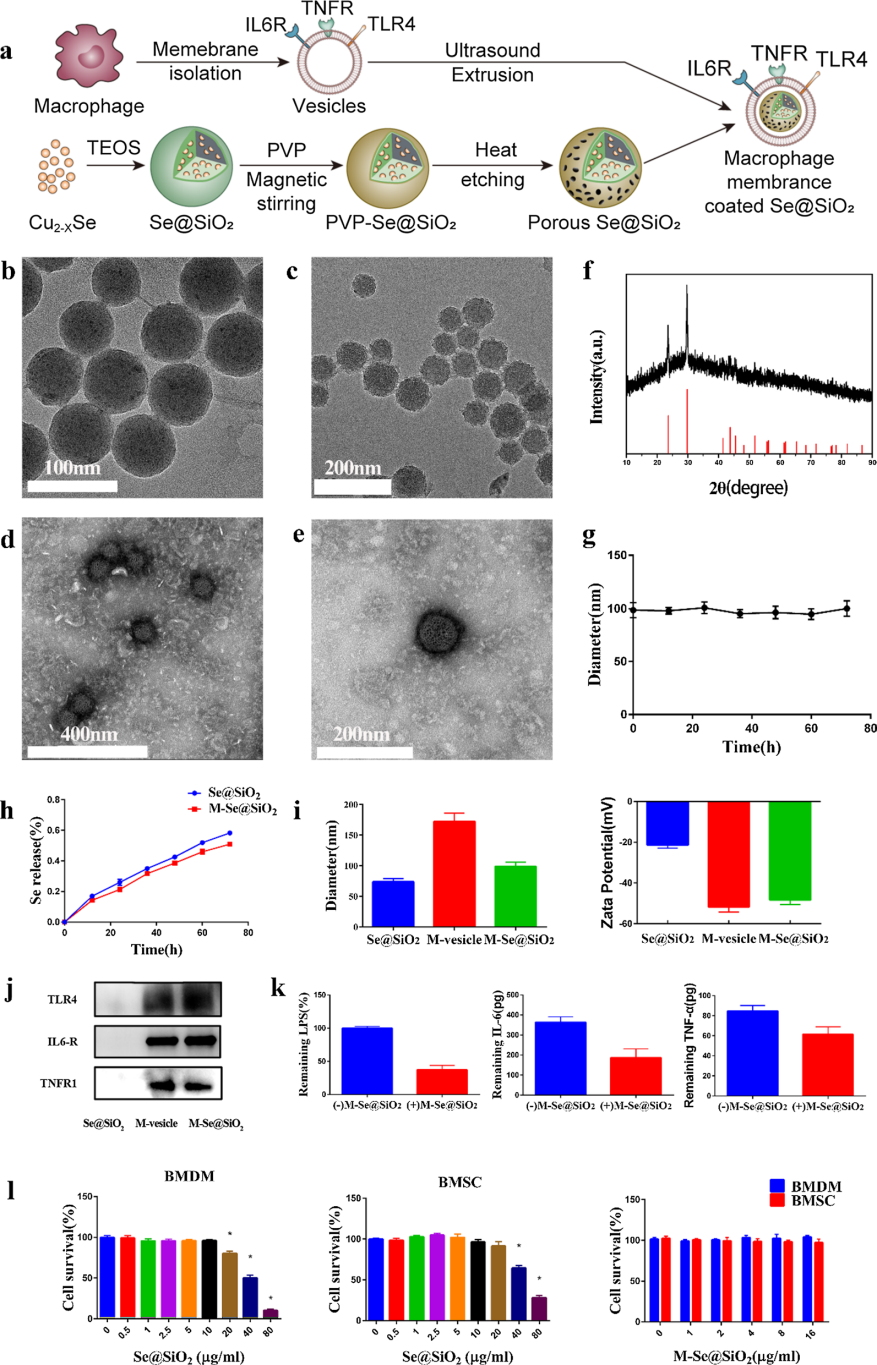
Characterization of macrophage membrane-coated porous Se@SiO_2_ nanospheres. **a** Schematic diagram of M-Se@SiO_2_ synthesis. TEM images of **b** Se@SiO_2_, **c** porous Se@SiO_2_ nanospheres, and **d**, **e** M-Se@SiO_2_. **f** X-ray diffractometry pattern of Se@SiO_2_ nanospheres and the standard Se hexagonal phase (JCPDS card No. 06–0362). **g** Hydrodynamic sizes of M-Se@SiO_2_ in PBS over 72 h. **h** Se release from M-Se@SiO_2_ and porous Se@SiO_2_ nanospheres in PBS at 37 °C and pH 7.4 over 72 h. **i** Size and zeta potential of porous Se@SiO_2_ nanospheres, M-vesicle, and M-Se@SiO_2_. **j** Western blots of TLR4, TNFR1, and IL6-R in porous Se@SiO_2_ nanospheres, M-vesicle, and M-Se@SiO_2_. **k** Removal of LPS and proinflammatory cytokines IL-6 and TNF-α by M-Se@SiO_2_. **l** Cell biocompatibility was evaluated using CCK-8 after 1 day of culture (*P < 0.05 compared with the control)

M-Se@SiO_2_ are composed of a macrophage membrane loaded onto the surface of porous Se@SiO_2_ nanospheres. After synthesizing porous Se@SiO_2_ nanospheres according to a previously described method [36] and isolating membrane vesicles from macrophages, the membrane vesicles were polymerized onto porous Se@SiO_2_ nanospheres (Fig. 1a). Transmission electron microscopy (TEM) images of the Se@SiO_2_ nanospheres showed abundant Se nanoparticles interspersed in the silica shell (Fig. 1b). Moreover, Se@SiO_2_ nanospheres formed porous structures after immersion in water (Fig. 1c). M-Se@SiO_2_ were subsequently dyed with phosphotungstic acid and examined by TEM. The nanoparticles showed a thin uniform coating on the surface (Fig. 1d, e). X-ray diffractometry (XRD) patterns of Se@SiO_2_ nanospheres corresponded well with the diffraction peaks of standard Se and showed a silica peak at ~ 23° (Fig. 1f), confirming the successful synthesis of Se@SiO_2_ nanospheres. There were no obvious changes in the hydrodynamic size of M-Se@SiO_2_ after 72 h (Fig. 1g), showing good colloidal stability. Due to the porous structure, Se could be continuously released from M-Se@SiO_2_ and porous Se@SiO_2_ nanospheres (Fig. 1h), thus confirming good biocompatibility and the potential for a long-term treatment effect. After membrane fusion, the diameter of the nanoparticles increased from ~ 73 to ~ 98 nm as measured by dynamic light scattering (DLS), which corresponded to a macrophage membrane thickness of approximately 12.5 nm on porous Se@SiO_2_ nanospheres. Moreover, the zeta potential of the membrane surface decreased from − 21.30 ± 1.61 to − 48.27 ± 2.34 mV, which is similar to that of macrophage membrane vesicles (Fig. 1i). In addition, western blotting was performed to analyze key proteins on the membrane surface. The receptor TLR4 is the main protein that binds to endotoxin, whereas IL6-R and TNFR bind to the pro-inflammatory factors IL-6 and TNF-α, respectively (Fig. 1j). These receptors were expressed on the macrophage membrane and exhibited strong binding potentials. Furthermore, the ability of M-Se@SiO_2_ to bind LPS and the proinflammatory cytokines TNF-α and IL-6 was investigated. Solutions containing LPS-fluorescein isothiocyanate (FITC) or cytokines were incubated with M-Se@SiO_2_ for 30 min, and the LPS or cytokine concentration was measured thereafter. M-Se@SiO_2_ effectively removed LPS, IL-6, and TNF-α, demonstrating that M-Se@SiO_2_ could effectively bind to LPS and pro-inflammatory cytokines (Fig. 1k). In summary, these results indicated that porous Se@SiO_2_ nanospheres were successfully modified with macrophage membranes that retained key proteins, thereby enhancing drug delivery, reducing toxin levels, and neutralizing inflammatory factors. The membranes used to coat nanospheres can play different roles depending on their cell of origin; for instance, erythrocytes with good immune evasion prolong nanosphere circulation, thereby extending the delivery of anti-infection drugs [23]. In contrast, tumor cells increase homologous recognition for antitumor therapy [24], and macrophages and neutrophils reduce immune system clearance while adsorbing endotoxins and cytokines to reduce inflammation [33, 37]. Indeed, platelet, bacterial, and mitochondrial membranes are increasingly used to coat nanospheres [31, 38, 39]. Moreover, the inner layer of porous Se@SiO_2_ nanospheres suppresses inflammation by releasing ultramicroscopic quantum dots of Se. Silica platforms are excellent nanocarriers owing to their high biocompatibility, controlled drug loading, and simple production [19]. Se, a trace element that regulates immune functions, has antioxidative properties, and promotes bone formation [40–42]. A study in a urethral wound healing model reported that porous Se@SiO_2_ nanospheres could regulate macrophage functions [43]. As expected, in the present study, M-Se@SiO_2_ inhibited LPS-induced osteolysis by both adsorption of proinflammatory mediators and regulation of macrophage polarization.

We used a Cell Counting Kit 8(CCK-8) assay to evaluate the cytotoxicity of porous Se@SiO_2_ nanospheres and M-Se@SiO_2_ to murine bone marrow-derived macrophages (BMDMs) and bone mesenchymal stem cells (BMSCs). Porous Se@SiO_2_ nanospheres did not show significant cytotoxicity up to 10 µg/mL. However, concentrations exceeding 10 µg/mL exhibited concentration-dependent cytotoxicity. Similarly, no significant cytotoxicity was detected after treatment with M-Se@SiO_2_ up to 10 µg/mL (Fig. 1l).

### In vitro evaluation of macrophage polarization

Inflammatory osteolysis caused by bacterial products or wear particles leads to bone loss near prosthesis. This is a major cause of joint replacement surgery failure [4, 44]. Macrophages are important immune cells that play a key role in osteolysis and are important targets of bone remodeling. Different polarization states of macrophages have distinct functions and can be influenced by specific external factors. M1-type macrophages express effector molecules, such as inducible nitric oxide synthase (iNOS); upregulate surface molecules, such as CD86 and C–C chemokine receptor type 7 (CCR7); and secrete proinflammatory cytokines, including IL-6 and TNF-α, which lead to inflammatory processes in osteolysis [6]. In contrast, M2-type macrophages secrete the anti-inflammatory factors IL-10 and IL-4 and express effector molecules, such as arginase 1 (ARG1), and surface molecules, such as CD206 and CD163 to inhibit inflammation and promote osteogenic differentiation and tissue regeneration [45].

**Fig. 2:**
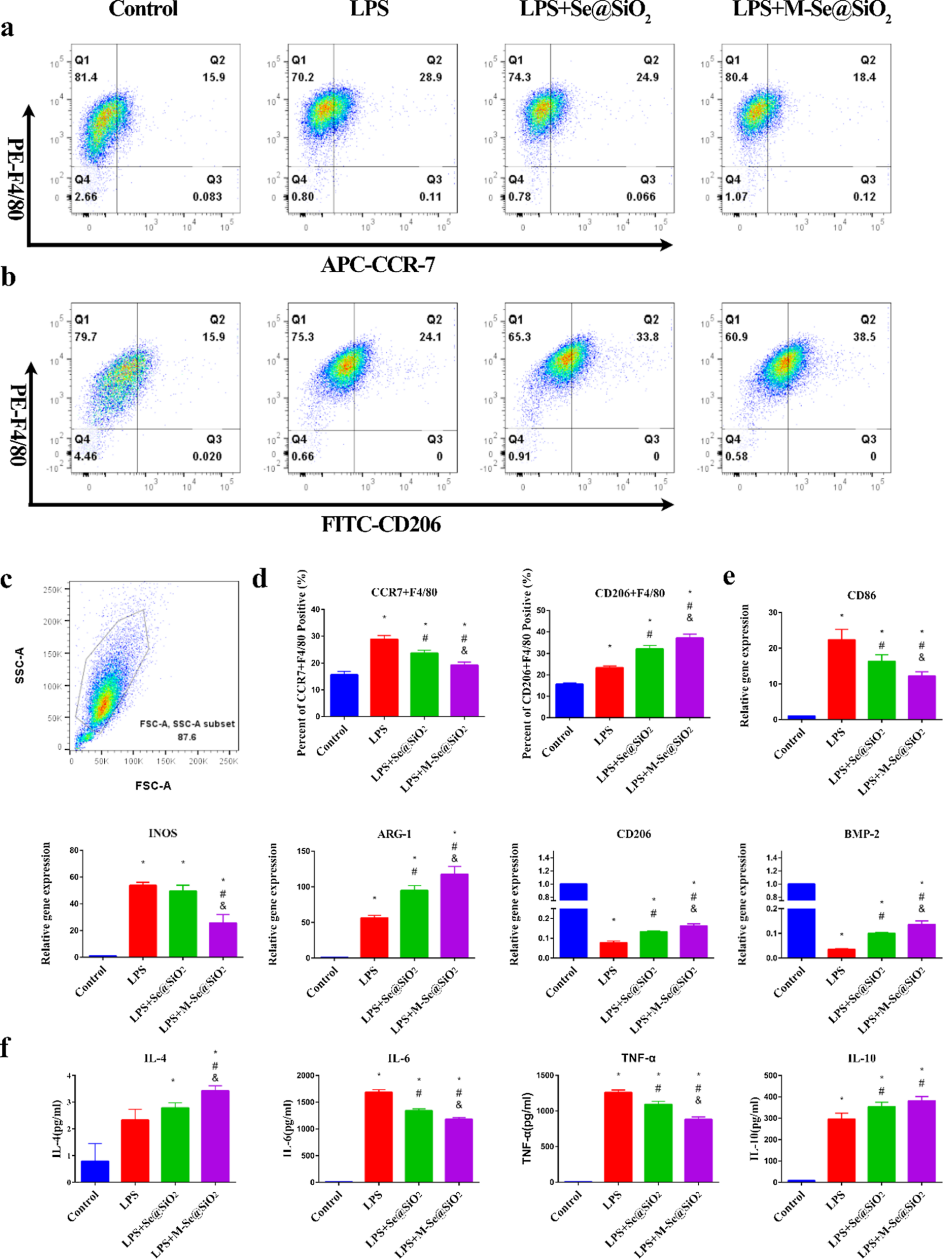
In vitro polarization of macrophages. **a**, **b** Representative dot plots of flow cytometry results after 24 h of culture of bone marrow-derived macrophages. Percentages of CCR7-, CD206-, and F4/80-positive cells. **c** Representative forward scatter (FSC) and side scatter (SSC) gates. **d** Proportions of CCR7 + F4/80 + and CD206 + F4/80 + cells. **e** RT-PCR results for *CD86*, *iNOS*, *ARG1*, *CD206,* and *BMP-2* expression. **f** ELISA results for IL-4, IL-6, TNF-α, and IL-10 (*, #, and & represent P < 0.05 compared with the control, LPS, and LPS + Se@SiO_2_, respectively)

**Fig. 3:**
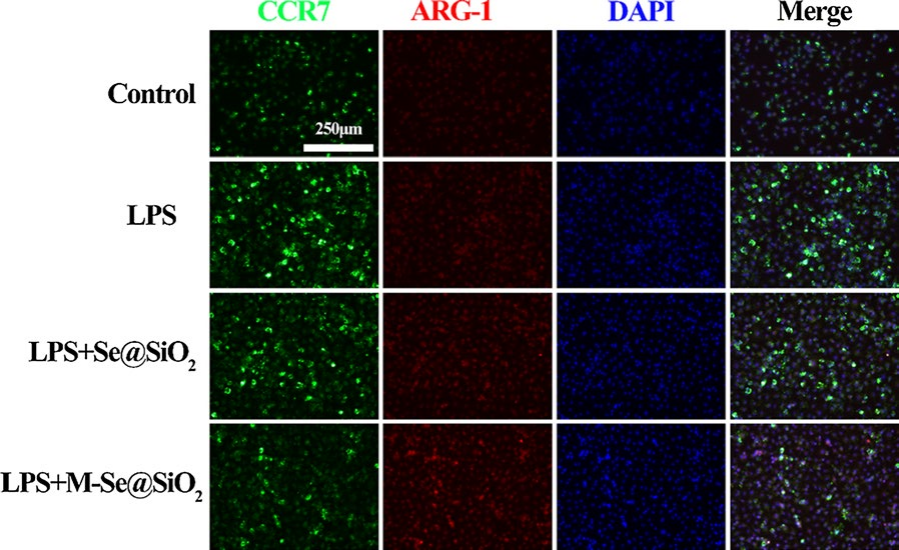
Immunofluorescence staining of BMDMs. CCR7 (green), ARG1 (red), and DAPI (blue; nuclei)

To identify macrophage phenotypes, we used flow cytometry to quantify the proportions of M1- and M2-type macrophages by evaluating the expression levels of CCR7 and CD206, surface markers for the M1 and M2 phenotypes, respectively (Fig. 2a–d). The percentage of CCR7-positive cells increased from 15.9% in the control group to 28.9% in the LPS-treated group. In contrast, CCR7 positivity decreased to 18.4% after M-Se@SiO_2_ treatment, and this proportion was lower than that in the Se@SiO_2_-treated group (24.9%). The percentage of M2 cells expressing the surface marker CD206 showed the following trend: control (15.9%) < LPS (24.1%) < LPS + Se@SiO_2_ (33.8%) < LPS + M-Se@SiO_2_ (38.5%). To investigate whether M-Se@SiO_2_ can regulate the M1/M2 polarization of BMDMs, we selected representative genes and assessed their expression using quantitative real-time polymerase chain reaction (RT-PCR) (Fig. 2e). The M1-associated genes *CD86* and *iNOS* were substantially downregulated in LPS-stimulated cultures treated with M-Se@SiO_2_ compared with LPS only-stimulated cultures, whereas the M2-associated genes *CD206* and *ARG-1* were upregulated. Furthermore, the osteoblast cytokine gene bone morphogenetic protein-2 (*BMP-2*) was upregulated in the LPS + M-Se@SiO_2_-treated group compared with the LPS-treated group. This suggests roles for M-Se@SiO_2_ in the immunomodulation occurring in osteogenesis and inhibition of inflammation. To further detect representative cytokines secreted by M1 and M2 macrophages, the concentrations of TNF-α, IL-6, IL-4, and IL-10 were measured using enzyme-linked immunosorbent assay (ELISA) (Fig. 2f). Cells treated with LPS + M-Se@SiO_2_ secreted greater amounts of the anti-inflammatory cytokines IL-4 and IL-10, which are mainly produced by M2 macrophages, than LPS- and LPS + Se@SiO_2_-treated macrophages. Moreover, macrophages treated with LPS + M-Se@SiO_2_ secreted less TNF-α and IL-6, proinflammatory factors produced by M1 cells, than those treated with LPS alone. Thus, the ELISA and PCR results agreed. The expression of the M1marker CCR7 (Alexa Fluor 488, green) and M2 marker ARG-1 (Alexa Fluor 594, red) in BMDMs was detected by immunofluorescence staining. The LPS + M-Se@SiO_2_ group exhibited fewer CCR7-positive cells than the LPS and LPS + Se@SiO_2_ groups. In contrast, the expression of ARG-1 was higher in the LPS + M-Se@SiO_2_ group than in the other groups (Fig. 3).

### M-Se@SiO_2_ attenuate LPS-induced activation of p65, ERK, and p38 phosphorylation in vitro

**Fig. 4:**
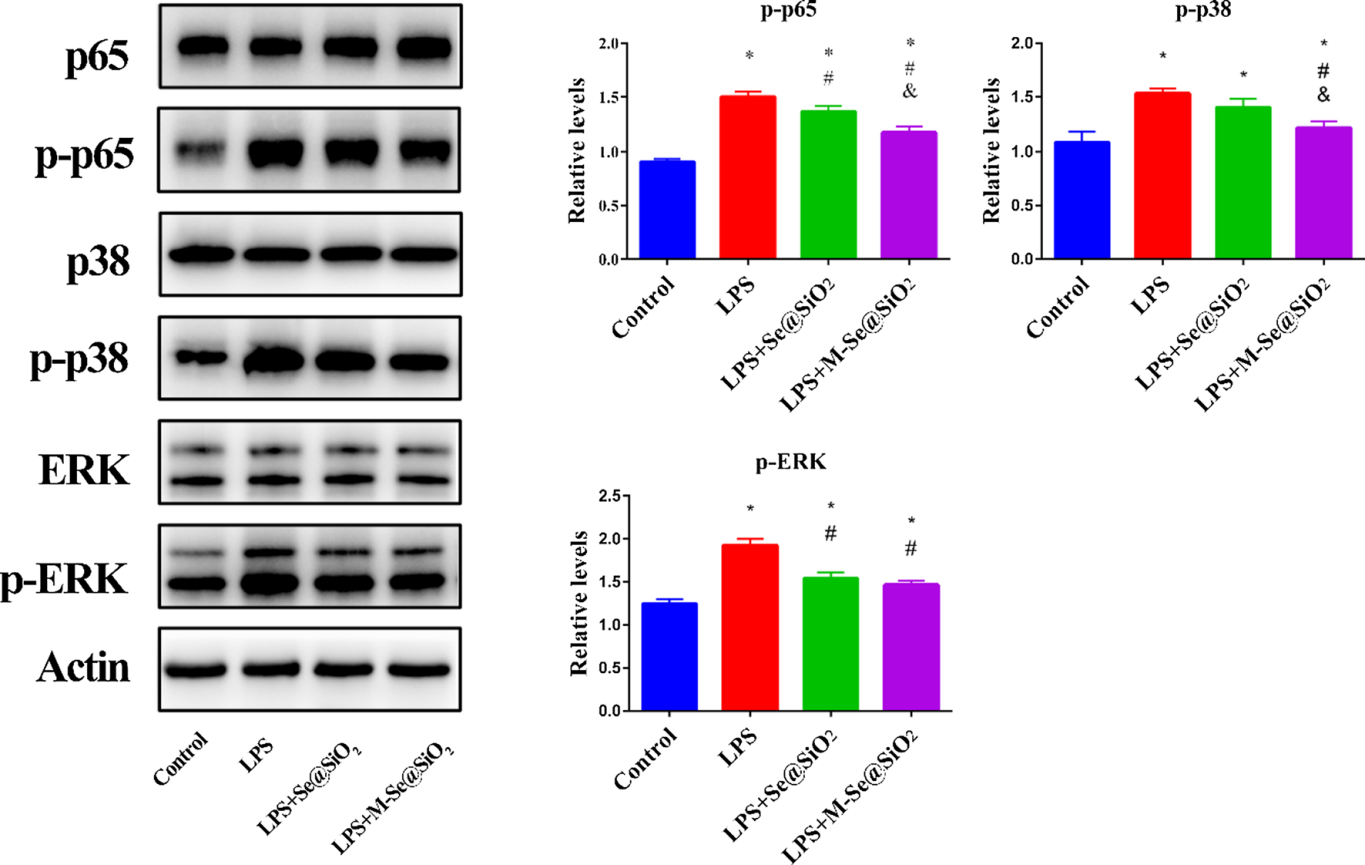
M-Se@SiO_2_ attenuates LPS-induced phosphorylation of p65, ERK, and p38 in BMDMs. **a** Western blots of macrophages treated with PBS (control), LPS, LPS + Se@SiO_2_, or LPS + M-Se@SiO_2_. **b**–**d** Relative quantification of the signal intensity of the western blot bands shown in **a** (*, #, and & represent P < 0.05 compared with the control, LPS, and LPS + Se@SiO_2_ groups, respectively)

LPS stimulation promotes M1 polarization via the TLR4 pathway. Generally, TLR4 mediates LPS stimulation downstream of the MAPK and NF-κB signaling pathways [46]. The NF-κB pathway is important in macrophage polarization. NF-κB is composed of homodimers or heterodimers (p65, p50) that bind to IκB in the cytoplasm when they are in their inactive state. Once activated, NF-κB p65 separates from IκB and translocates to the nucleus, where it binds to the promoters of its target genes [47]. The MAPK pathway is also a key pathway in macrophage polarization and inflammation, involving the ERK, p38, and c-Jun N-terminal kinase (JNK) subfamilies. Activation of the ERK, p38, and JNK pathways in response to LPS stimulation leads to phosphorylation of these proteins and promotes the expression of proinflammatory factors. Therefore, both NF-κB and MAPK are ideal targets for anti-inflammatory drugs [22]. Moreover, Se can inhibit the MAPK and NF-κB pathways, thereby exerting anti-inflammatory effects [48]. To investigate the effects of M-Se@SiO_2_ treatment on the LPS-stimulated NF-κB pathway, the abundance of p65 was examined. Significant inhibition of LPS-induced p65 phosphorylation was observed after M-Se@SiO_2_ administration. LPS also activates the MAPK pathway and drives M1 macrophage polarization. In the present study, LPS promoted the phosphorylation of p38 and ERK, whereas M-Se@SiO_2_ treatment inhibited their phosphorylation (Fig. 4). Taken together, our results suggest that M-Se@SiO_2_ inhibit LPS-induced polarization of M1 macrophages through the MAPK/NF-κB signaling pathways.

### Osteogenic differentiation capacity of BMDM-conditioned medium

**Fig. 5:**
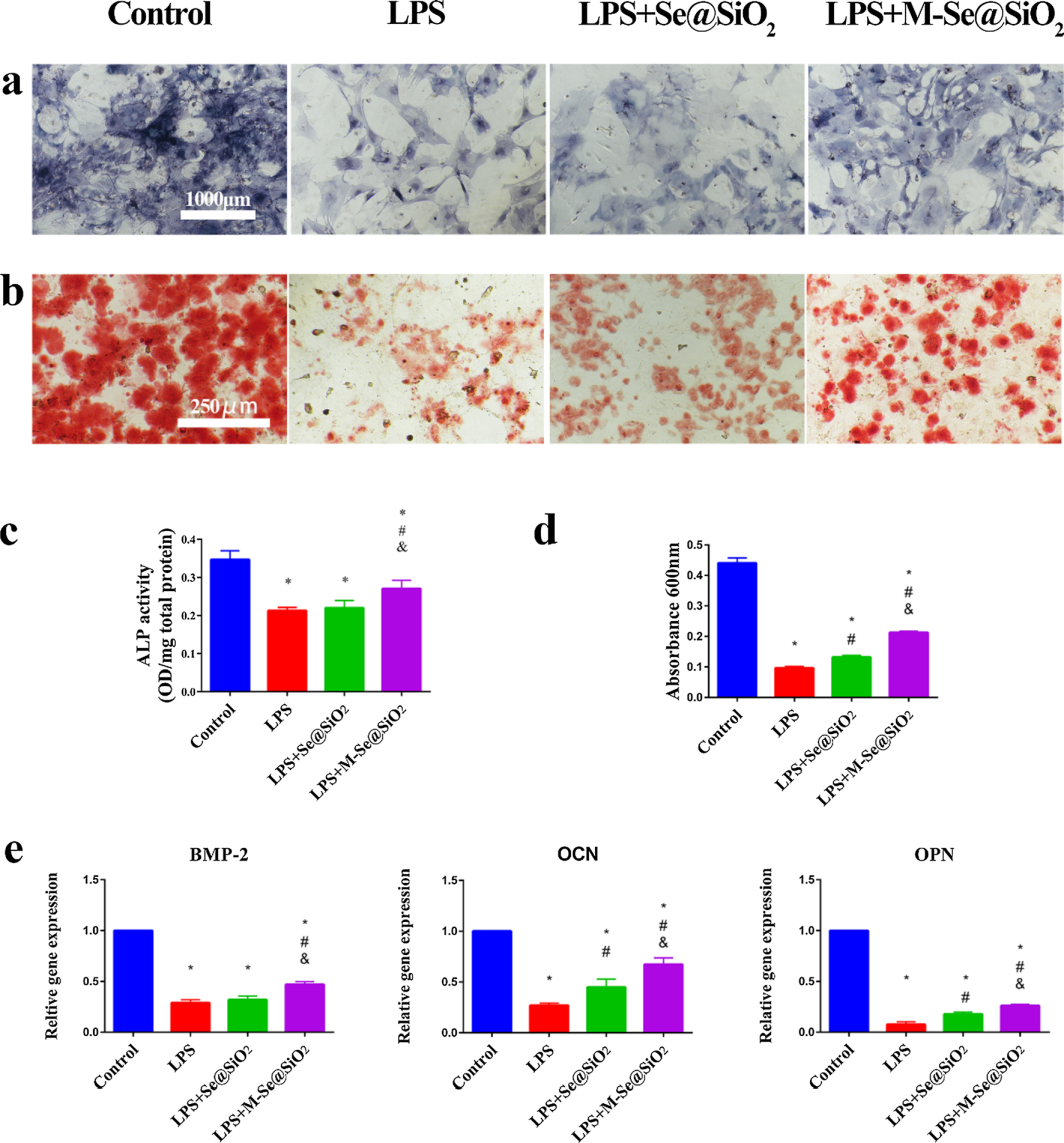
Osteogenic differentiation induced by macrophage-conditioned medium. **a** ALP and **b** ARS staining of BMSCs cultured in conditioned medium for 14 days. **c** ALP activity of BMSCs cultured in conditioned medium. **d** Quantitative analysis of ARS staining. **e** Osteogenesis-related gene expression of BMSCs cultured in conditioned medium (*, #, and & represent P < 0.05 compared with the control, LPS, and LPS + Se@SiO_2_ groups, respectively)

Previous studies have shown that macrophages play a central regulatory role in all phases of bone regeneration and that cytokines contribute to their effects [12]. Therefore, we prepared conditioned media by collecting macrophage culture supernatants to investigate the effects of macrophage-derived cytokines on the osteogenic process. BMDM cell culture supernatant was used as a conditioned medium to assess the effects of secreted factors from these macrophages on the osteogenic differentiation of BMSCs. The results of alkaline phosphatase (ALP) and Alizarin Red (ARS) staining are shown in Fig. 5a and b. ALP and ARS staining was weaker in the LPS group than in the control group, indicating the inhibition of osteogenic differentiation. In contrast, the staining intensities were stronger in the LPS + M-Se@SiO_2_ group than in the LPS + Se@SiO_2_ and LPS groups. Quantification of ALP and ARS straining revealed similar results (Fig. 5c, d). We also examined the expression of three crucial genes related to osteogenesis, *BMP*-2, osteocalcin (*OCN*), and osteopontin (*OPN*) by RT-PCR. All genes were downregulated following incubation with LPS-conditioned medium compared with control treatment. Additionally, the LPS + M-Se@SiO_2_ group presented higher mRNA expression of these three genes than the LPS and LPS + Se@SiO_2_ groups (Fig. 5e). These findings indicate that the osteogenic process was significantly inhibited in the presence of conditioned medium after LPS stimulation, whereas osteogenic differentiation was upregulated after M-Se@SiO_2_ treatment compared with LPS or LPS + Se@SiO_2_ treatment.

### In vivo air pouch and cranial bone models

**Fig. 6:**
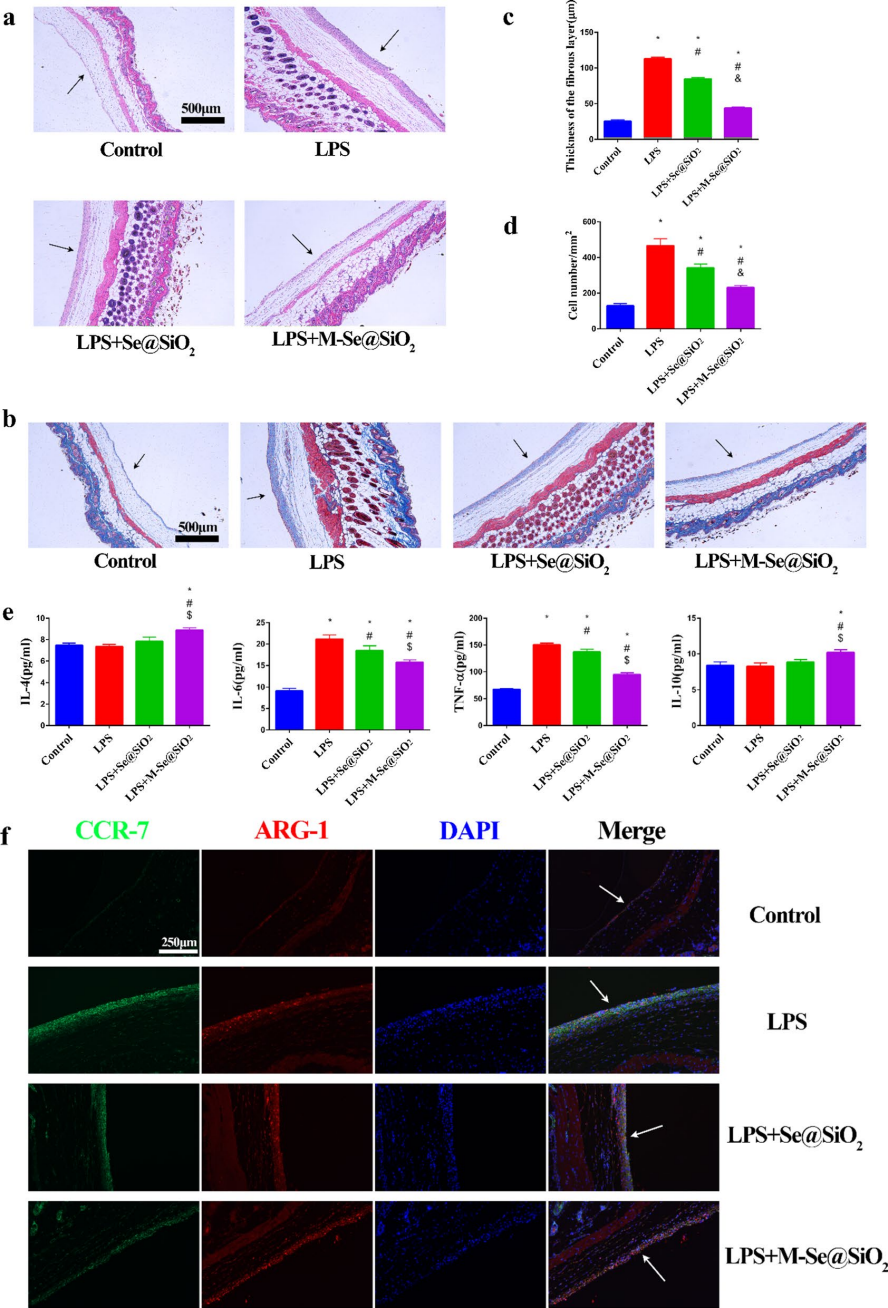
Staining of air pouch tissues on day 4. **a** H&E and **b** Masson trichrome staining of skin tissue from air pouches. Arrows point to the fibrous layer. **c** Thickness of the fibrous layer. **d** Numbers of infiltrating cells. Fibrous layer thickness and inflammatory cell infiltration were decreased in the LPS + M-Se@SiO2 group compared with the LPS and LPS + Se@SiO2 groups. **e** Cytokines in air pouch exudates evaluated by ELISA. **f** Immunofluorescence staining of skin tissue from air pouches. CCR7 (green), ARG1 (red), and DAPI (blue; nuclei). Arrows point to the fibrous layer (*, #, and & represent P < 0.05 compared with the control, LPS, and LPS + Se@SiO_2_ groups, respectively)

The mouse air pouch model was used to assess the immunomodulatory effect of M-Se@SiO_2_ on the inflammatory response and phenotypes of macrophages. Sterile air was injected subcutaneously into mice to form an air pouch. As shown in Fig. 6a and b, hematoxylin and eosin (H&E) and Masson trichrome staining of air sac tissues from LPS-treated mice showed increases in fibrous layer thickness and inflammatory cell infiltration. Fibrous layer thickness and inflammatory cell infiltration were decreased in the LPS + M-Se@SiO_2_ group compared with the LPS and LPS + Se@SiO_2_ groups (Fig. 6c, d). Inflammatory exudates were used for cytokine detection by ELISA. The abundances of the proinflammatory cytokines TNF-α and IL-6 were lower in the LPS + M-Se@SiO_2_ group than in the LPS group, whereas those of the anti-inflammatory cytokines IL-4 and IL-10 were highest in the LPS + M-Se@SiO_2_ group, similar to the in vitro results (Fig. 6e). Immunofluorescence staining further demonstrated that the fibrous layer contained more CCR7-positive cells in the LPS group than in the control, LPS + Se@SiO_2_, and LPS + M-Se@SiO_2_ groups. In contrast, the number of ARG1-positive cells was higher in the LPS + M-Se@SiO_2_ group than in the other groups, indicating that M-Se@SiO_2_ induced M2-type polarization and an anti-inflammatory response (Fig. 6f). These findings indicated that M-Se@SiO_2_ effectively inhibited LPS-induced inflammatory responses by regulating macrophage polarization.

**Fig. 7:**
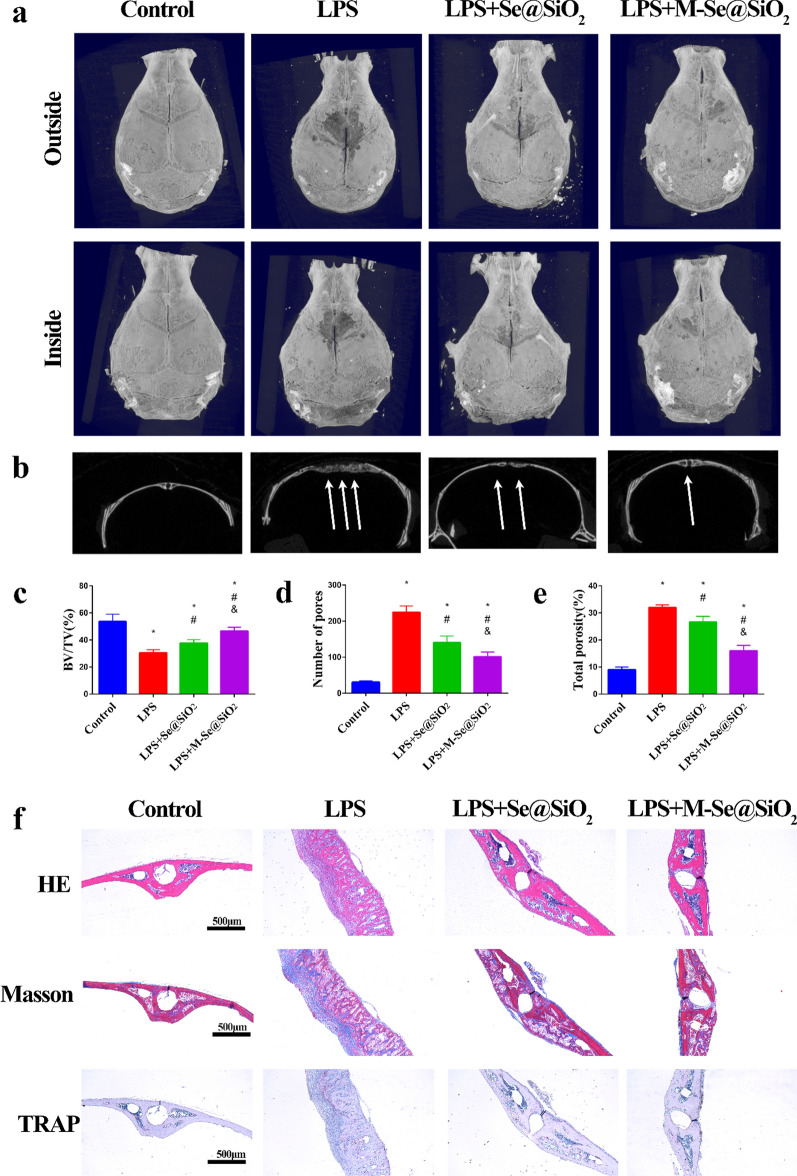
M-Se@SiO_2_ attenuates LPS-induced osteolysis in vivo. Representative micro-CT **a** 3D- and **b** 2D-reconstructed images of the calvaria in each group. White arrows indicate osteolysis. **c** The BV/TV, **d** number of pores, and **e** total porosity in each group were measured using micro-CT analyzer software. **f** Histological images of H&E-stained, Masson trichrome-stained, and TRAP-stained calvarium sections in each experimental group (*, #, and & represent P < 0.05 compared with the control, LPS, and LPS + Se@SiO_2_ groups, respectively)

After confirming that M-Se@SiO_2_ have the potential to regulate macrophage polarization, we evaluated the potential of M-Se@SiO_2_ to protect against LPS-induced inflammatory osteolysis using an in vivo cranial bone model established in mice. Fourteen days after surgery, micro-computed tomography(micro-CT) analyses revealed increased cranial bone destruction and osteolysis in the LPS-treated group compared with the control group (Fig. 7a, b). However, the treatment with M-Se@SiO_2_ significantly inhibited osteolysis and bone resorption. Furthermore, quantitative analysis of morphometric parameters revealed that M-Se@SiO_2_ treatment significantly inhibited the reduction in bone volume (BV/TV, ratio of bone volume to tissue volume) and increased bone porosity after LPS-treatment (Fig. 7c–e). Histological examination further confirmed the protective effects of M-Se@SiO_2_ against LPS-induced osteolysis. H&E and Masson’s trichrome staining revealed extensive bone resorption and inflammatory cell infiltration in the LPS group compared with the other groups, whereas the M-Se@SiO_2_ group showed significantly less bone destruction and inflammation. Tartrate-resistant acid phosphatase (TRAP) staining showed that TRAP-positive cells were obviously induced by LPS and that the number of TRAP-positive cells was decreased in the M-Se@SiO_2_ treatment group. Collectively, our results suggest that treatment with M-Se@SiO_2_ is protective against LPS-induced osteolytic bone loss in vivo (Fig. 7f).

In vivo biocompatibility was further evaluated in mice at 14 days after cranial surgery. H&E staining of the heart, liver, lungs, spleen, and kidneys confirmed nontoxicity (Additional file 1: Figure S1). The mouse stain studied showed excellent biocompatibility with the nanoparticles, indicating the potential of these nanoparticles for clinical application.

## Conclusions

Our findings suggest that M-Se@SiO_2_ play an immunomodulatory role in LPS-induced inflammation and bone remodeling. The results of our in vitro and in vivo experiments suggest that M-Se@SiO_2_ inhibit the polarization of macrophages toward the M1 phenotype and reduce the release of proinflammatory factors while increasing the levels of anti-inflammatory, and osteogenic, factors to suppress inflammation and reduce osteolysis. At the molecular level, this effect might be mediated through the regulation of the NF-κB and MAPK signaling pathways. In conclusion, M-Se@SiO_2_ are promising engineered nanoparticles for the treatment of osteolysis arising after arthroplasty and have potential for use in the further development of immunomodulatory nanoplatforms.

## Materials and methods

### Macrophage membrane derivation

Murine macrophage RAW264.7 cells, kindly provided by Stem Cell Bank (Chinese Academy of Sciences, China), were cultured in Dulbecco’s modified Eagle’s medium (HyClone, USA) containing 10% fetal bovine serum (FBS; Gibco, Australia) and 1% penicillin/streptomycin (Gibco, USA) at 37 °C in 5% CO_2_. The cell membrane was extracted from RAW264.7 cells using a membrane protein extraction kit (Beyotime, China). First, the cells were immersed for 15 min in ice-cold membrane protein extraction reagent; then, the cells were moderately disrupted using a Dounce homogenizer. Nuclei and a small number of unbroken cells were removed by low-speed centrifugation (700 × *g*, 10 min), and the supernatant was subjected to high-speed centrifugation (14,000 × *g*, 30 min) to obtain the cell membrane precipitate. The membrane protein content was measured using a BCA kit (Beyotime). To obtain macrophage membrane vesicles, an ultrasound bath (42 kHz, 100 W) was used. Ultrasound was applied to cell membranes for 15 min, followed by 11 extrusions using an Avanti mini extruder with 400 nm polycarbonate porous membranes (Avanti, Canada) [34].

### M-Se@SiO_2_ preparation and characterization

Porous Se@SiO_2_ nanospheres were synthesized as previously reported [36]. First, Cu_2−x_Se nanocrystals were oxidized to form Se quantum dots. Solid Se@SiO_2_ nanospheres were formed by coating silica onto Se quantum dots by orthosilicate hydrolysis in an alkaline environment. Next, the solid Se@SiO_2_ nanospheres were coated with polyvinylpyrrolidone and treated with hot water to form porous structures. TEM (TF20; FEI, USA) and XRD (Rigaku, Japan) were used for nanoparticle detection and characterization. The collected macrophage membranes and porous Se@SiO_2_ nanospheres were mixed at a 1:1 mass ratio of membrane proteins to nanoparticles using the ultrasound bath for 3 min. The membranes were then passed through 100-nm polycarbonate porous membranes 11 times using an Avestin mini extruder to obtain M-Se@SiO_2_. M-Se@SiO_2_ were detected by negative staining for TEM. Briefly, 3 μL of nanoparticle suspension (1 mg/mL) was deposited on a copper grid and subsequently stained with 1 wt% phosphotungstic acid. Samples were then observed using a Talos 120 kV Sphera microscope. DLS was used to measure the size and zeta potential of the nanoparticles. M-Se@SiO_2_ at 1 mg/mL were mixed with 2 × phosphate-buffered saline (PBS) at a 1:1 volume ratio, and nanoparticle stability in PBS was assessed. Se release from porous Se@SiO_2_ nanospheres and M-Se@SiO_2_ was studied separately using a Leeman ICP-AES Prodigy instrument as previously described [49].

### LPS and cytokine neutralization

To evaluate the LPS- and cytokine-binding ability of M-Se@SiO_2_, M-Se@SiO_2_ (1 mg/mL) were mixed with PBS containing 10% FBS and FITC-LPS (Sigma, 100 ng/mL), TNF-α (85 pg/mL), or IL-6 (360 pg/mL) at 37 °C for 30 min. The samples were centrifuged at 16,000 × *g* for 15 min to remove the nanospheres. LPS remaining in the supernatant was measured as the fluorescence intensity, and cytokines in the supernatant were quantified by ELISA (Anogen, Canada).

### Characterization of membrane proteins

Membrane proteins from macrophages were extracted using a membrane protein extraction kit (Beyotime), and the abundance of cell membrane surface proteins, including TLR4, TNFR1, and IL6-R, were detected by western blotting.

### BMDM cultures

The femurs of C57/BL6 mice were washed with α-modified Eagle’s medium (α-MEM; Gibco, USA) to obtain bone marrow cells. The extracted cells were maintained in complete α-MEM containing 10% FBS (Gibco, Australia), 1% penicillin/streptomycin (Gibco, USA), and 40 ng/mL macrophage colony-stimulating factor (Pepro Tech, USA) at 37 °C in 5% CO_2_ for 7 days to obtain mouse BMDMs.

### Cell biocompatibility assay

Cell viability was analyzed using a CCK-8(Dojindo, Japan). BMDMs were seeded in 24-well plates at a density of 5 × 10^5^ per well, while BMSCs were plated at a density of 1 × 10^5^ per well. After 24 h of incubation with different concentrations of porous Se@SiO_2_ nanospheres or M-Se@SiO_2_, the cells were cultured in medium containing 10% CCK-8 for 2 h at 37 °C. The absorbance of the samples was then read at 450 nm using a microplate reader (BioTek, USA).

### Immunofluorescence staining

Immunofluorescence staining for the M1-like macrophage marker CCR7 and M2-like macrophage marker ARG1 was performed to assess macrophage polarization. BMDMs were divided into four groups: control (vehicle-treated cells; PBS, used for the dissolution of LPS), LPS (100 ng/mL), LPS + porous Se@SiO_2_ nanospheres (10 µg/mL; Se@SiO_2_ group) and LPS + M-Se@SiO_2_ (10 µg/mL; M-Se@SiO_2_ group). After 24 h of culture, the BMDMs were fixed in paraformaldehyde (4%), blocked with Blocking Buffer for Immunol Staining (Beyotime, China) for 15 min, and incubated with a mouse anti-CCR7 (1:100, Abcam, USA) or rabbit anti-ARG1 (1:100, Abcam, USA) antibody overnight at 4 °C. The next day, the cells were washed and incubated with a donkey anti-mouse Alexa Fluor 594-conjugated (1:200, Abcam, USA) or donkey anti-rabbit Alexa Fluor 488-conjugated (1:200, Abcam, USA) antibody for 1 h at room temperature protected from light. The cells were then washed with PBS, and the nuclei were stained with 4ʹ,6-dimidazole-2-phenylindole (DAPI) for 5 min. Images were acquired using a DM8 microscope (Leica, USA).

### Flow cytometry

Flow cytometry was carried out to analyze the abundance of the M1 marker CCR7, M2 marker CD206, and general macrophage marker F4/80. After 24 h of culture, BMDMs in the four groups were scraped, washed, blocked for 15 min with Blocking Buffer (Beyotime), and then stained for 1 h with an allophycocyanin (APC)-conjugated anti-CCR7 antibody (1:100, BioLegend, USA) or FITC-conjugated anti-CD206 antibody (1:100, BioLegend, USA). A PE-conjugated anti-F4/80 antibody (1:100, BioLegend, USA) was used to label all macrophages. The cells were analyzed using a BD flow cytometer with FlowJo software.

### ELISA

After 24 h of incubation, culture medium was collected from BMDMs in the four groups and centrifuged. Cytokine levels (TNF-α, IL-4, IL-6, and IL-10) in the supernatant were determined using ELISA kits (Anogen, Canada) according to the manufacturer’s instructions.

### RT-PCR

After 24 h of incubation, total RNA was extracted from BMDMs in the four groups using an RNA extraction column kit (EZBioscience, USA) according to the manufacturer’s instructions. Subsequently, complementary DNA was synthesized from 1 μg of total RNA using a cDNA synthesis kit (EZBioscience, USA). Quantitative RT-PCR was performed using a SYBR Green Master mix (EZBioscience, USA) on LightCycler 480 (Roche, USA). The primers used are shown in Additional file 1: Table S1.

### Western blotting

BMDMs were pretreated with porous Se@SiO_2_ nanospheres (Se@SiO_2_ group; 10 µg/mL) or M-Se@SiO_2_ (M-Se@SiO_2_ group; 10 µg/mL) for 1 h, and then LPS was added (100 ng/mL). After 30 min, the cells were lysed with RIPA lysis buffer containing protease and phosphatase inhibitors (EpiZyme, China). The extracted total proteins were separated by sodium dodecyl sulfate–polyacrylamide gel electrophoresis and subsequently transferred onto polyvinylidene fluoride membranes. The membranes were blocked in a blocking solution (Beyotime) for 15 min, washed, and incubated with primary antibodies overnight at 4 °C. Antibodies specific for p38/p-p38, ERK/p-ERK, and p65/p-p65 (1:1000, Cell Signaling, USA) were used in the experiments. After washing, the membranes were incubated with a goat anti-rabbit IgG horseradish peroxidase-conjugated antibody (1:1000, Cell Signaling, USA) for 1 h at room temperature. The blots were developed with an enhanced chemiluminescent reagent (Millipore, USA) and the Tanon Imaging System (Tanon, China) for chemiluminescent detection. Relative band intensities were quantified using ImageJ software, and all results were normalized to β-actin expression.

### BMSC cultures and conditioned medium preparation

Primary mouse BMSCs were isolated as previously described [4] and cultured in α-MEM containing 10% FBS and 1% penicillin/streptomycin. After culturing BMDMs for 24 h, the culture medium was collected from the control, LPS, LPS + Se@SiO_2_ and LPS + M-Se@SiO_2_ groups and centrifuged; the resulting supernatants were then mixed with osteogenic induction medium (Cygen, China) at a ratio of 1:2 to obtain conditioned media. Next, BMSCs were seeded in 24-well plates at a density of 2.5 × 10^4^ cells per well. Following cell adherence, the culture medium was replaced with the conditioned medium, which was subsequently changed every 2 days.

### ALP and ARS staining

BMSCs were fixed in 4% paraformaldehyde and stained with an ALP (Beyotime, China) or ARS (Cytogen, China) dye after 14 days of culture. ALP staining was quantified using an ALP assay kit (Beyotime, China) according to the manufacturer’s instructions. Quantitative analysis of ARS staining was performed by adding 10% acetylchlorinated pyridine to release ARS after ARS staining and subsequently measuring the optical density at 600 nm.

### Analysis of gene expression in BMSCs exposed to BMDM-conditioned medium

After 2 weeks of incubation, the expression of the osteogenesis-related genes of *BMP-2*, *OCN*, and *OPN* in BMSCs was analyzed by RT-PCR. The primers used are shown in Additional file 1: Table S1.

### In vivo air pouch model established in mice

The animal experiments were approved by the Animal Care Committee of Shanghai Jiao Tong University Affiliated Sixth People’s Hospital. C57BL/6 mice were used in the experiments as described previously [5]. After mildly anesthetizing mice using pentobarbital, 5 mL of sterile air was injected subcutaneously. Four days after air pouch formation, 0.5 mL of PBS or 0.5 mL of PBS with 1 µg/mL LPS was injected with or without porous Se@SiO_2_ nanospheres or M-Se@SiO_2_. Four days after injection, the mice were sacrificed, and exudates obtained by washing the air pouches with 2 mL of PBS were centrifuged and stored at − 80 °C for ELISA analysis. Finally, the air pouch tissue was collected and fixed with 4% paraformaldehyde. The tissue sections were subjected to H&E or Masson trichrome staining to assess inflammation, immune cell infiltration, and membrane thickness. Immunofluorescence staining was used to detect CCR7- and ARG1-positive cells in the air pouch tissue. Images were captured using a DM6 microscope (Leica, USA) and analyzed using ImageJ software.

### In vivo calvarial osteolysis model establish in mice

Mice were anesthetized with intraperitoneal pentobarbital injections. A 1-cm long incision was made in the middle of the cranium, and the cranial periosteum was separated from the calvarium. Next, 50 µL of LPS (1 mg/mL) was embedded under the periosteum around the sagittal midline suture of the calvaria. In the experimental groups, PBS, porous Se@SiO_2_ nanospheres, or M-Se@SiO_2_ were injected intraperitoneally at a dose of 0.5 mg/kg every 3 days. The animals were sacrificed 14 days after surgery. The cranial bones were carefully harvested, fixed in 4% paraformaldehyde, and stored in 70% ethanol until they were imaged with a micro-CT scanner (Bruker micro-CT) at a resolution of 18 mm. The BV/TV, total porosity, and number of pores were measured and analyzed using CT Analyser Software (Bruker) as previously described [50]. Before paraffin embedding, the bones were decalcified in 14% EDTA, pH 7.4, for 2 weeks. Then, H&E, Masson trichrome and TRAP staining was performed. Stained sections were imaged using a DM6 microscope (Leica, USA). In addition, the heart, kidneys, liver, spleen and lungs of mice were collected and fixed to assess the biosafety of the administered nanoparticles in major organs. Sections of these major organs were embedded in paraffin, cut, and stained with H&E.

### Statistical analysis

Data are presented as the mean ± standard deviation and were analyzed using SPSS v. 18.0 software (SPSS Inc., Chicago, IL, USA). Statistical significance among groups was evaluated using the one-way analysis of variance and a *t*-test. Results with p < 0.05 were considered statistically significant.

## Supplementary Information

**Additional file 1: Figure S1**. Evaluation of the biocompatibility of each agent with major organs. H&E staining shows excellent biocompatibility in in vivo experiments evaluating the nanoparticles. **Figure S2**. Representative image of an SDS gel showing protein bands corresponding to different nanoparticles. **Figure S3**. Quantification of relative CCR7 and ARG-1 fluorescence in images, related to Figure 3 (*, #, and & represent P < 0.05 compared with the control, LPS, and LPS+Se@SiO2 groups, respectively). **Figure S4**. Quantification of relative CCR7 and ARG-1 fluorescence in images, related to Figure 6f (*, #, and & represent P < 0.05 compared with the control, LPS, and LPS+Se@SiO2 groups, respectively). **Table S1**. Primers for RT-PCR used to quantify expression in BMDMs and BMSCs.

## Data Availability

All study data are included in this article.

## Abbreviations

Se@SiO_2_: SiO_2_-coated ultrasmall Se particles
M-Se@SiO_2_: Macrophage membrane-coated porous Se@SiO_2_ nanospheres
LPS: Lipopolysaccharide
ERK: Extracellular signal-regulated kinase
IL: Interleukin
TNF-α: Tumor necrosis factor-alpha
TLR4: Toll-like receptor 4
MAPK: Mitogen-activated protein kinase
TEM: Transmission electron microscopy
XRD: X-ray diffractometry
DLS: Dynamic light scattering
CCK-8: Cell Counting Kit 8
BMDM: Bone marrow-derived macrophage
BMSC: Bone mesenchymal stem cell
iNOS: Inducible nitric oxide synthase
CCR7: C–C chemokine receptor type 7
ARG1: Arginase 1
RT-PCR: Real-time polymerase chain reaction
BMP2: Bone morphogenetic protein-2
ELISA: Enzyme-linked immunosorbent assay
NF-κB: Nuclear factor-κB
JNK: C-Jun N-terminal kinase
ALP: Alkaline phosphatase
ARS: Alizarin Red
OCN: Osteocalcin
OPN: Osteopontin
BV/TV: Ratio of bone volume to tissue volume
TRAP: Tartrate-resistant acid phosphatase
PBS: Phosphate-buffered saline
α-MEM: α-Modified Eagle’s medium
FBS: Fetal bovine serum
DAPI: 4ʹ,6-Dimidazole-2-phenylindole
FITC: Fluorescein isothiocyanate
H&E: Hematoxylin and eosin
