# Supplementary material for: Macrophage-biomimetic porous Se@SiO2 nanocomposites for dual modal immunotherapy against inflammatory osteolysis
Source: J Nanobiotechnology. 2021 Nov 22;19:382. doi: 10.1186/s12951-021-01128-4 (PMC8607681; doi:10.1186/s12951-021-01128-4)
Supplement: Supplementary file 1 — Additional file 1: Figure S1. Evaluation of the biocompatibility of each agent with major organs. H&E staining shows excellent biocompatibility in in vivo experiments evaluating the nanoparticles. Figure S2. Representative image of an SDS gel showing protein bands corresponding to different nanoparticles. Figure S3. Quantification of relative CCR7 and ARG-1 fluorescence in images, related to Figure 3 (*, #, and & represent P < 0.05 compared with the control, LPS, and LPS+Se@SiO2 groups, respectively). Figure S4. Quantification of relative CCR7 and ARG-1 fluorescence in images, related to Figure 6f (*, #, and & represent P < 0.05 compared with the control, LPS, and LPS+Se@SiO2 groups, respectively). Table S1. Primers for RT-PCR used to quantify expression in BMDMs and BMSCs. [file 12951_2021_1128_MOESM1_ESM.docx]

Supporting Information


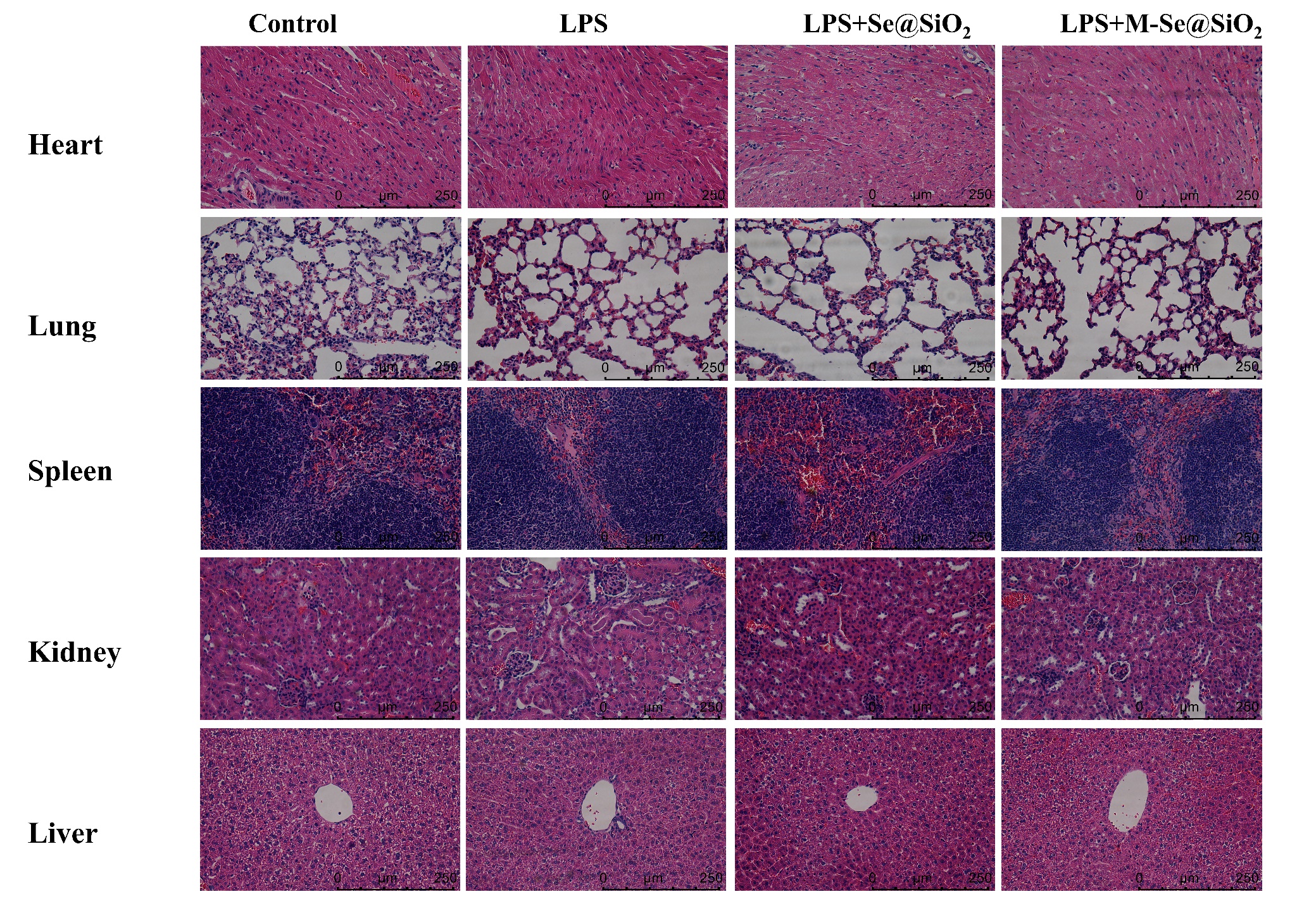


**Figure S1.** Evaluation of the biocompatibility of each agent with major organs. H&E staining shows excellent biocompatibility in *in vivo* experiments evaluating the nanoparticles.


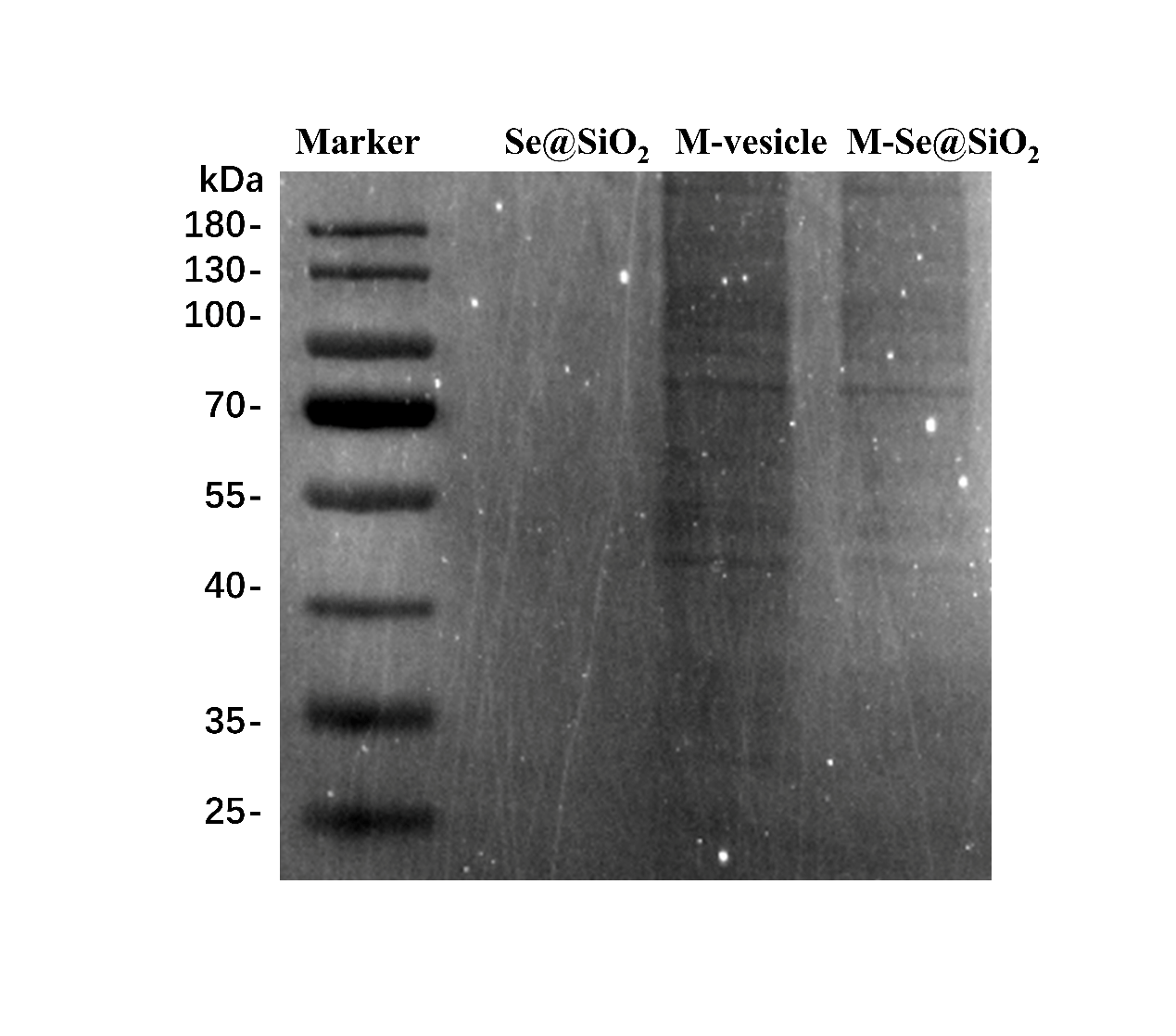


**Figure S2.** Representative image of an SDS gel showing protein bands corresponding to different nanoparticles


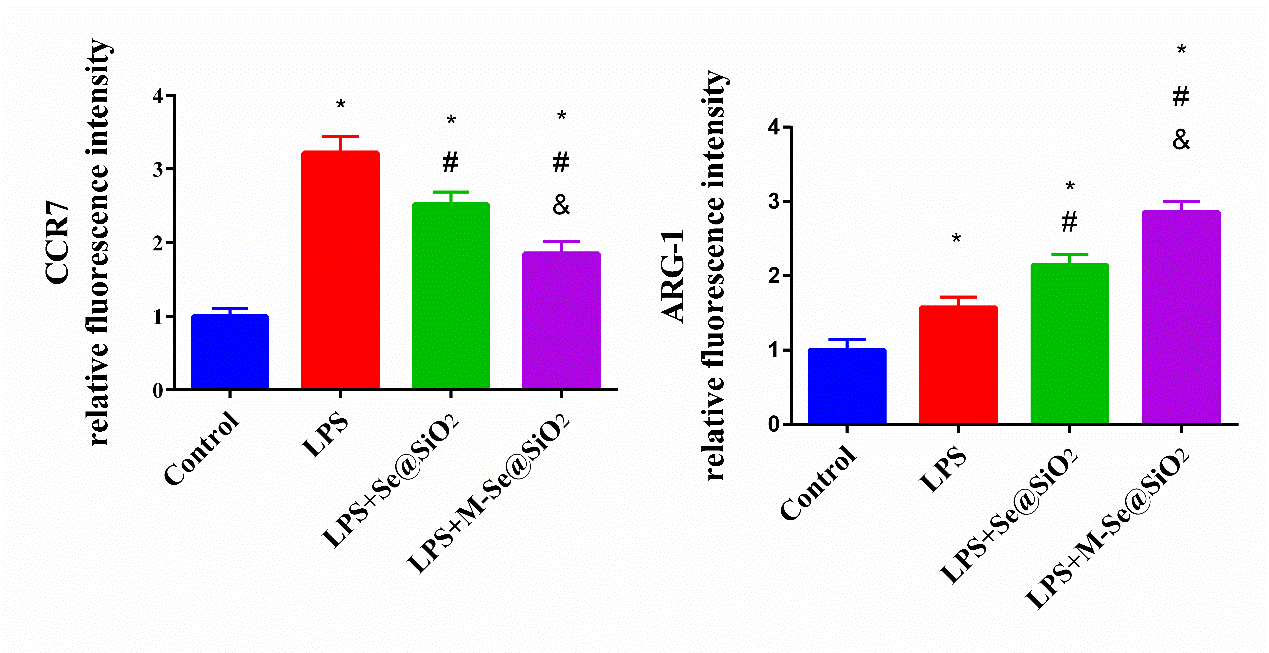


**Figure S3.** Quantification of relative CCR7 and ARG-1 fluorescence in images, related to Figure 3 (*, #, and & represent P < 0.05 compared with the control, LPS, and LPS+Se@SiO2 groups, respectively).
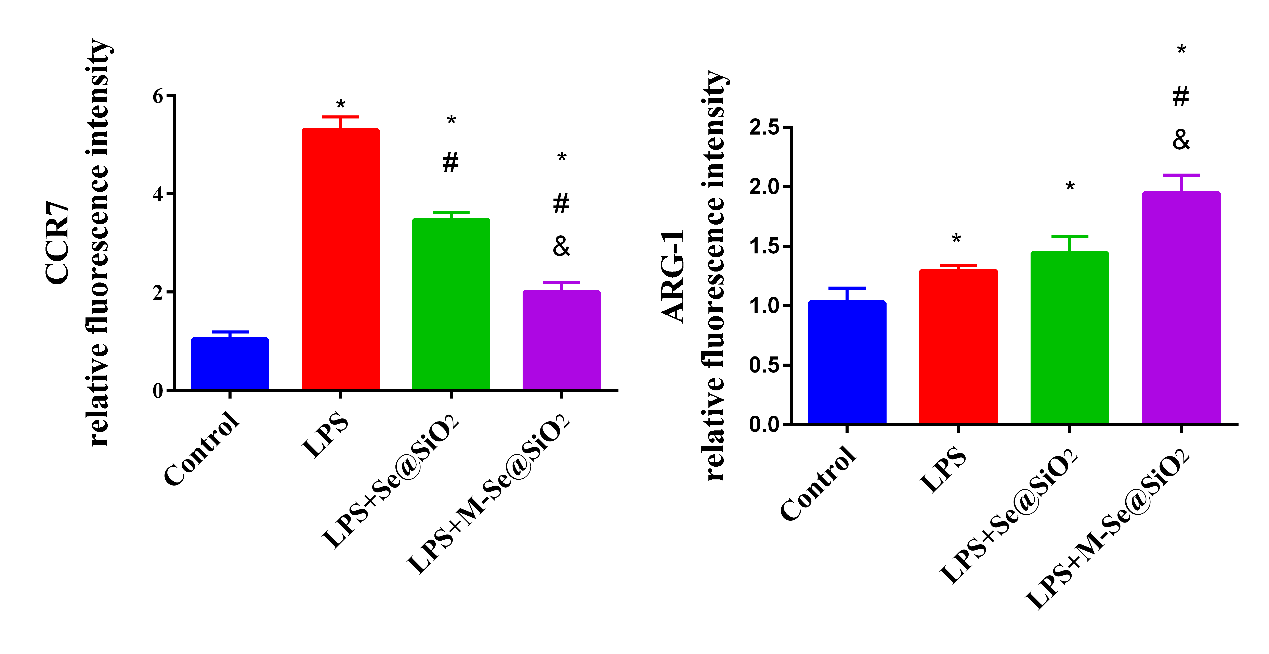


**Figure S4.** Quantification of relative CCR7 and ARG-1 fluorescence in images, related to Figure 6f (*, #, and & represent P < 0.05 compared with the control, LPS, and LPS+Se@SiO2 groups, respectively).

| Gene | Primer sequences (5’ to 3’) |
| --- | --- |
| **CD86** | **F:**ACGGAGTCAATGAAGATTTCCT  **R**:GATTCGGCTTCTTGTGACATAC |
| **iNOS** | **F:**CACCTTGGAGTTCACCCAGT  **R**:ACCACTCGTACTTGGGATGC |
| **CD206** | **F:**CCTATGAAAATTGGGCTTACGG  **R**:CTGACAAATCCAGTTGTTGAGG |
| **ARG-1** | **F:**CARGGGCAACCTGTGTCCTT **R**:TCCTGGTACATCTGGGAACTTTC |
| **BMP-2** | **F:**AGTAGTTTCCAGCACCGAATTA  **R**:CACTAACCTGGTGTCCAATAGT |
| **OCN** | **F:**ACCGCCTACAAACGCATCTA **R**:AGAGGACAGGGAGGATCAAGT |
| **OPN** | **F:**CTTGAGCATTCCAAAGAGAGC **R**:CTTGTGGCTGTGAAACTTGTG |
| **β-actin** | **F:**CTACCTCATGAAGATCCTGACC  **R**:CACAGCTTCTCTTTGATGTCAC |

**Table S1.** Primers for RT-PCR used to quantify expression in BMDMs and BMSCs.
